# Continuous Subcutaneous Delivery of Proline-Rich Antimicrobial Peptide Api137 Provides Superior Efficacy to Intravenous Administration in a Mouse Infection Model

**DOI:** 10.3389/fmicb.2019.02283

**Published:** 2019-10-02

**Authors:** Daniel Knappe, Rico Schmidt, Knut Adermann, Ralf Hoffmann

**Affiliations:** ^1^Faculty of Chemistry and Mineralogy, Institute of Bioanalytical Chemistry, Leipzig University, Leipzig, Germany; ^2^Center for Biotechnology and Biomedicine, Leipzig University, Leipzig, Germany; ^3^AMP-Therapeutics GmbH, Leipzig, Germany

**Keywords:** apidaecin, continuous subcutaneous delivery, *E. coli* ATCC 25922, intraperitoneal infection, systemic septicaemia

## Abstract

Apidaecins are cationic, proline-rich antimicrobial peptides originally isolated from honeybees and exhibit high Gram-negative activity by inhibiting bacterial protein translation. Pharmacokinetics of apidaecin derivative Api137 was studied using single and multiple intravenous or subcutaneous injections as well as continuous subcutaneous infusion and correlated to its efficacy in a lethal murine *Escherichia coli* infection model. Survival rates of infected CD-1 mice were monitored and Api137 and its metabolites were quantified in plasma of uninfected CD-1 mice and Sprague Dawley rats using reversed-phase chromatography coupled online to mass spectrometry. The highest Api137 plasma levels of 23 mg/L were obtained after a single intravenous injection of 20 mg/kg body weight, which declined fast over the next 120 min (half-life time < 30 min). In contrast, continuous subcutaneous infusion of a similar dose over an hour (19.2 mg/kg/h) lead to stable plasma levels of ∼6 mg/L, which was above the minimal inhibitory concentration against *E. coli* ATCC 25922 (4 mg/L). The increased exposure by continuous subcutaneous administration of Api137 at 19.2 mg/kg/h over 48 h improved efficacy in the murine intraperitoneal sepsis model with survival rates of 67% over 5 days compared to 33% after intravenous and subcutaneous administration in different dosing schemes. To the best of our knowledge, continuous subcutaneous infusion using osmotic pumps was successfully utilized for delivery of an antimicrobial peptide for the first time. Additionally, the potential of apidaecin analogs as novel antibiotics is demonstrated even in a scenario where the infection site is clearly separated from the route of administration.

## Introduction

Antimicrobial peptides (AMP) are expressed in a variety of organisms and represent promising alternatives to current antibiotics (Fox, 2013; Czaplewski et al., 2016). Peptide-based antibiotics share favorable *in vitro* properties of other peptide therapeutics, such as high efficacy and selectivity to various target molecules, low tissue enrichment, and low toxicity, while exhibiting disadvantages, such as low metabolic stability, fast renal clearance, and low oral availability. However, new treatment options for infections by multi-resistant bacteria are urgently needed to circumvent entry into a post-antibiotic era.

Antimicrobial peptides representing novel antimicrobial lead compounds can be separated into two groups by their general mechanism of action, either showing direct membrane-active or lytic effects, or by entry into bacterial cells, binding and inhibiting specific intracellular targets (Lohner and Hilpert, 2016; Scocchi et al., 2016).

In particular, cationic proline-rich antimicrobial peptides (PrAMPs) belong to the second group binding to the negatively charged bacteria surface by electrostatic interactions through basic amino acid residues. Proline residues account for extended, partially polyproline helix II-like secondary structures that most likely support free penetration through the outer membrane of Gram-negative bacteria. PrAMPs accumulate in the periplasmic space where the transporters SbmA and MdtM actively translocate them into the cells (Mattiuzzo et al., 2007; Runti et al., 2013; Krizsan et al., 2015a). Internalized PrAMPs bind to chaperone DnaK (Otvos et al., 2000; Liebscher et al., 2010) and 70S ribosomes (Krizsan et al., 2014; Roy et al., 2015; Seefeldt et al., 2015, 2016; Gagnon et al., 2016) and inhibit protein translation *in vitro* and *in vivo* (Krizsan et al., 2014, 2015b; Mardirossian et al., 2014). In more detail, interactions of PrAMPs with 70S ribosome and ribosomal proteins can be differentiated to oncocin- and apidaecin-type binding modes. Insect-derived oncocin, pyrrhocoricin and metalnikowin as well as mammalian Bac7(1-16) bind inside the peptide exit tunnel of matured 70S ribosome of *Thermus thermophilus* and interfere with the initiation step of translation (Roy et al., 2015; Seefeldt et al., 2015, 2016; Gagnon et al., 2016). In contrast, apidaecin Api137 binds also to matured 70S ribosomes of *E. coli* as indicated by fluorescence polarization, but inhibits *in vitro* protein translation only slightly (Krizsan et al., 2014). However, it inhibits expression of green fluorescent protein in vital *E. coli* cells as efficient as oncocins. Sedimentation experiments indicate inhibition of the 50S subunit assembly as likely mechanism/pathway (Krizsan et al., 2015b). As a second mechanism, Api137 binds to fully assembled bacterial ribosomes trapping the release factors RF1 and RF2 subsequently to the release of the nascent protein (Florin et al., 2017; Matsumoto et al., 2017). Both, apidaecins and oncocins are well tolerated in mice after intraperitoneal (ip) injection of daily doses up to 320 mg/kg and 160 mg/kg body weight (BW), respectively (Schmidt et al., 2016, 2017). Only one mouse showed a very weak immune response with a very low level of anti-Api137 IgG antibodies when Api137 was weekly administrated ip (10 mg/kg BW) for 8 weeks (Holfeld et al., 2015). Analogs of both PrAMP families are efficacious *in vivo* in different murine infection models of intraperitoneal sepsis with *E. coli* ATCC 25922, KPC-2-producing *Klebsiella pneumoniae* K 97/09, and intramuscular thigh infection with *K. pneumoniae* ATCC 43816 (Knappe et al., 2015; Schmidt et al., 2016, 2017). In mouse PK studies after ip and iv administration at 5 mg/kg BW, oncocin Onc72 and Onc112 plasma levels were determined at 1.8 and 5.4 mg/L (ip), and 5.7 and 8.4 mg/L (iv) (Schmidt et al., 2016). Both peptides were rapidly eliminated during the following 90 min primarily by renal clearance as indicated by high peptide concentrations of 40–100 μg/g in kidney homogenates (Schmidt et al., 2016). The plasma levels of apidaecins Api88 and Api137 were significantly lower reaching only 1.0 and 0.4 mg/L after iv and 0.6 and 0.2 mg/L after ip administration of 5 mg/kg BW (Schmidt et al., 2017). This is surprising as apidaecin Api137 is most efficacious among the four tested PrAMPs in an intraperitoneal sepsis model with *E. coli* ATCC 25922 (Schmidt et al., 2017).

Here, the pharmacokinetics of Api137 are studied after iv and subcutaneous (sc) administration with doses of 5, 10, and 20 mg/kg BW in mice and rats. Both administration routes were evaluated to achieve protection of mice intraperitoneally infected with *E. coli* ATCC 25922. In a second part of the program, ALZET^®^ osmotic pump techniques are optimized and applied for continuous sc delivery of apidaecin analog Api137 to achieve constant blood levels over a longer period and correlate PK with pharmacodynamic parameters in an infection model.

## Materials and Methods

Api134 (Gu-ONNRPVYIPRPRPPHPOL-NH_2_; Gu: *N*,*N*,*N′*,*N′-*tetramethylguanidino, O: L-ornithine) and Api137 (Gu-ONNRPVYIPRPRPPHPRL-OH) were provided as peptide acetate salts from CBL Patras (Patras, Greece). Api137 containing ^13^C_5_,^15^N-L-proline in position 16 was synthesized in house as described recently (Schmidt et al., 2017).

### Alzet^®^ Osmotic Pumps

ALZET^®^ mini-osmotic pump Model 2001D (length 3.0 cm, diameter 0.7 cm, total displaced volume 1.0 mL; DURECT Corporation, Cupertino, CA, United States) were utilized for continuous sc administration. The pump had a fill volume of 200 μL (varies from batch to batch in acceptable limits) and the expected pumping rate was 8 μL/h for at least 24 h (192 μL/24 h). Api137 was completely dissolved at concentrations of 20, 40, and 60 mg/mL in 0.9% saline within one minute and filled into the pump according to the manufacturers protocol. Thus, pumps supposedly delivered 6.4, 12.8, and 19.2 mg/kg/h corresponding to daily doses of 154, 307, and 461 mg/kg, respectively, considering a body weight of 25 g. After filling, pumps were primed in 0.9% saline (10 mL) 37°C for at least 3 h according to the manufacturers protocol. Due to the higher viscosity of the higher concentrated peptide solutions, the priming time had to be extended to 6 h and constant pump rate was checked by transferring the pumps to fresh prewarmed tubes containing saline (10 mL) at selected intervals. The peptide was quantified in the solution by reversed phase chromatography. Delivery of the peptide solution was additionally approved by removing the residual volume from the pump according to the manufacturers protocol.

### Animals

All animal studies were performed at ViviSource Laboratories, Inc. (Waltham, MA, United States) in compliance with the Animal Welfare Act, the Guide for the Care and Use of Laboratory Animals, and the Office of Laboratory Animal Welfare (approved Institutional Animal Care and Use Committee, IACUC protocols No. VVS12-026 and VVS12-030). Female CD-1 mice and female Sprague Dawley (SD) rats were obtained from Charles River Laboratory (Wilmington, MA) and acclimated in randomized groups for at least 5 days before use. Standard rodent diet (LabDiet 5001, PharmaServ, Framingham, MA, United States) and water were given *ad libitum*. Mice were housed six per cage and one per cage after ALZET^®^ pump implantation in a room maintained at 20–26°C with humidity set at 40–70% and a 12-h light-dark cycle.

### Tolerance Study

Eight female CD-1 mice (22–30 g, *n* = 2) or four SD rats (218–260 g, *n* = 1) were treated iv (10 and 5 mL/kg, respectively) with Api137 formulated in 0.9% saline (TEKnova, Hollister, CA, United States) at concentrations of 5, 10, 25, and 50 mg/kg body weight and their behavior monitored for 24 h.

### Pharmacokinetics

Api137 dissolved in 0.9% saline was injected iv or sc in mice (18–26 g, total = 51) and rats (204–248 g, total = 12) at a dose volume of 10 and 5 mL/kg BW, respectively, according to the dosing scheme (Supplementary Figures 1, 2). Three mice per dose and time point were euthanized by carbon dioxide inhalation and blood was collected via cardiac puncture into K_2_-EDTA Microtainer^®^ tubes (Becton Dickinson and Company, BD, Franklin Lakes, NJ, United States). Additionally, blood was collected via saphenous vein puncture from three rats into identic tubes.

After priming, as described above, pumps were implanted under isoflurane anesthesia into a pocket of the subcutaneous connective tissue between the scapula of the mice. The pump was inserted with the flow moderator pointing away from the incision to the tail. Three animals per dose were bled twice via submandibular puncture and euthanized at the third time point by carbon dioxide inhalation and cardiac puncture (Supplementary Table 1). EDTA-blood samples were inverted for 10 min and centrifuged (3000 × *g*, 5 min, 4°C). Plasma was transferred to polypropylene tubes and stored at −80°C prior to analysis.

### Infection Model

*Escherichia coli* ATCC 25922 was obtained from American Type Culture Collection (Manassas, VA, United States). Cells were grown overnight on solid agar, suspended in saline and adjusted to an OD of 0.1 at 625 nm. The suspension was further diluted in 5% hog gastric mucin (Sigma-Aldrich Corporation, St. Louis, MO, United States) and final inoculums between 4.8 × 10^5^ and 2.0 × 10^6^ colony forming units (cfu) per mouse were injected ip (500 μL). Api137 and levofloxacin was dissolved in sterile saline and sterile water, respectively, and were administered iv (5 mL/kg) into the tail vein or sc (10 mL/kg) into the back. Alternatively, an hour post infection ALZET^®^ osmotic pumps were implanted under isoflurane anesthesia (see above). After 24 h, pumps were exchanged once and finally removed after 48 h. Animals were observed for 7 days post infection.

### Quantification of Api137

Api137 was quantified in EDTA plasma samples using reversed-phase chromatography (RP-HPLC) coupled to electrospray ionization mass spectrometry (ESI-MS/MS) at TNO Triskelion (Zeist, Netherlands). Analog Api134 was used as internal standard. Briefly, internal standard solution (10 μL, 5 mg/L) was added to a plasma sample (50 μL) and mixed with aqueous phosphoric acid (50 μL; 4%, v/v). Solid phase extraction was performed on a WCX micro-elution plate (Waters Corporation, Milford, MA, United States) with a final elution using aqueous acetonitrile (75%, v/v) containing 1% (v/v) trifluoroacetic acid. Extracts were analyzed on an Acquity C18 BEH column (2.1 mm internal diameter, 10 cm length, 1.7 μm particle size; Waters) using an Acquity UPLC system coupled online to a XEVO TQS mass spectrometer (Waters; for details see Supplementary Material). Alternatively, Api137 and metabolites 1–16 and 1–17 were quantified for selected samples in house as reported recently (Schmidt et al., 2017).

### Statistical Analysis

The log-rank (Mantel-Cox) test was used for comparison of the survival curves and dose-response curves were fitted with the “dose-response log(inhibitor) vs. normalized response – variable slope” function with “least squares fit” using GraphPad Prism 5.02.

## Results

### Tolerance After Intravenous Injection

No adverse effects were observed after iv administration of Api137 at a dose of 5 mg/kg in mice and rats. At doses of 10 and 20 mg/kg mice became lethargic within minutes directly after dosing with piloerection for the next 45 min but recovered to normal limits within 1 h. Mice that received 50 mg/kg Api137 iv, experienced convulsion and low blood pressure immediately after dosing and died within 1 min. Rats treated with 10 to 50 mg/kg of Api137 experienced labored breathing and lethargy but recovered to normal limits within 5 h post injection.

### Pharmacokinetics With Intravenous and Subcutaneous Injection

**FIGURE 1:**
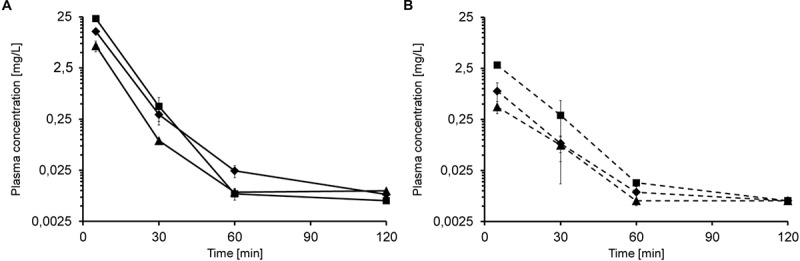
Plasma concentration of Api137 in CD-1 mice after single dose iv (**A**; full lines) and sc (**B**; dashed lines) injection of 5 (▲), 10 (◆), and 20 mg/kg BW (■), respectively. Each data point represents the mean of peptide concentration in plasma of three individual mice at a logarithmic scale.

Based on the tolerance data, single iv injections of 5, 10, and 20 mg/kg led to dose-dependent plasma levels of 6.8, 12.9, and 23.2 mg/L after 5 min, respectively (Figure 1A). After 30 min, the plasma levels decreased to 0.09 mg/L, 0.30 mg/L and 0.44 mg/L, respectively. Api137 was still detected after 60 min in all mice at around 0.01 mg/L (lower limit of quantification, LLOQ = 5 μg/L). The plasma levels obtained 5 min after single sc injections of 5, 10, and 20 mg/kg were significantly lower with 0.4, 0.9 and 2.9 mg/L, respectively, but still dose-dependent (Figure 1B). After 30 min, the concentrations decreased to 0.08 mg/L (5 and 10 mg/kg) and 0.3 mg/L (20 mg/kg). For the highest dose, Api137 was still detected after 60 min (0.014 mg/L).

**FIGURE 2:**
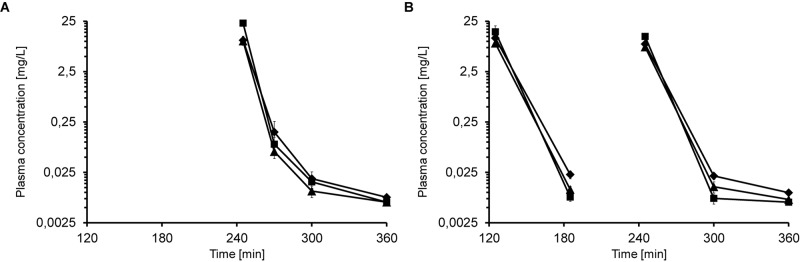
Plasma concentrations of Api137 in CD-1 mice after two (**A**; 0 and 240 min) and three (**B**; 0, 120, and 240 min) consecutive iv injections at doses of 5 (▲), 10 (◆), and 20 mg/kg BW (■), respectively. Each data point represents the mean peptide concentration of three plasma obtained from individual mice. Peptide concentrations are given at logarithmic scale.

When mice received multiple iv injections of Api137, the peptide did not accumulate in the plasma. Five minutes after the second iv injection of 5, 10, and 20 mg/kg, 245 min after the first administration, the plasma concentrations of 10.1, 10.5, and 22.9 mg/L, respectively, were comparable to a single injection (Figure 2A). Thirty minutes after the second injection (time point 270 min) plasma levels decreased to 0.06, 0.16, and 0.09 mg/L (5–20 mg/kg) and were still above the LOQ after 300 min (0.01–0.02 mg/L).

The third dosing scheme consisted of three iv injections at 0, 2 and 4 h (Supplementary Figure 1). Plasma levels of the low and medium doses at 5 min post second and third injection (time points 185 min and 245 min, 7.7–11.5 mg/L) were similar to the corresponding values obtained after one and two injections (Figure 2B). In contrast, plasma levels of the highest dose group were lower (15.4 and 12.5 mg/L) than obtained for one and two administrations (23.2 and 22.8 mg/L). For medium and low dose groups, Api137 was detected in all animals at all later time points at concentrations slightly above LOQ (0.005–0.02 mg/L), whereas the plasma levels in the highest dose group drop partially below LOQ.

In Sprague Dawley rats the peak plasma levels were not dose-dependent after 5 min ranging from 4.0 to 9.8 mg/L for three dose groups and therefore twofold lower than determined in mice (Supplementary Figure 3A). Thirty minutes after iv administration the plasma levels were 0.16–0.21 mg/L at a comparable range as seen for mice. Single sc injections of 5, 10, and 20 mg/kg resulted in low dose-dependent levels of 0.1, 0.4, and 0.8 mg/L, respectively, after 5 min (Supplementary Figure 3B). After 30 min, the concentrations decreased approximately threefold to 0.03, 0.08, and 0.25 mg/L, respectively. After 60 min, Api137 was detected for the highest dose only (0.02 mg/L). Similar to mice, peptide Api137 did not accumulate in rats after multiple injections (Supplementary Figure 4).

### Intraperitoneal Infection Treated by iv and sc Administration of Api137

**FIGURE 3:**
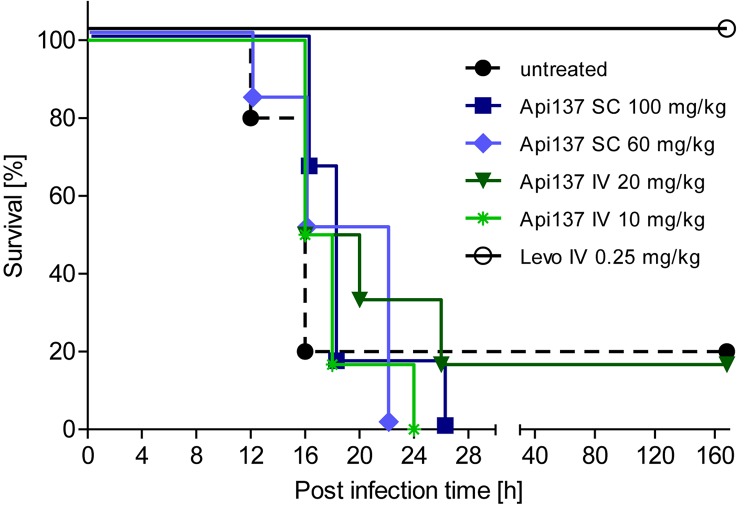
Survival of female CD-1 mice (25–28 g, total = 42) infected ip with *E. coli* ATCC 25922 (2.0 × 10^6^ cfu/mouse) for different treatment strategies. Api137 was administered iv at 10 mg/kg (light green, asterisks) and 20 mg/kg (dark green, triangles) or sc at 60 mg/kg (light blue, diamonds) and 100 mg/kg (dark blue, squares) at 1, 4, 8, and 12 h post infection (*n* = 6). Levofloxacin was administered iv at 1 h post infection (black, open circles, *n* = 6) while the control group remained untreated (black dashed line, filled circles, *n* = 12).

Infection of CD-1 mice with 5 × 10^5^ to 2 × 10^6^ cfu *E. coli* ATCC 25922 (ip) typically leads to severe signs of illness, and untreated mice had to be euthanized after 12–16 h. Two treatment schemes using Api137 with two (1 and 5 h post infection) or four consecutive intravenous injections (30, 60, 90, and 120 min post infection) did not rescue the animals (Supplementary Figures 5, 6). Using a longer treatment period and four injections at 1, 4, 8, and 12 h post infection, did also not significantly increase the survival rates after 48 h. However, a weak therapeutic effect was obvious from the longer survival times with the mean survival time of animals receiving 60 and 100 mg/kg sc or 20 mg/kg iv doses of Api137 increasing by 3 h (Figure 3 and Supplementary Table 2).

### Pharmacokinetics With Continuous Subcutaneous Infusion

As an alternative treatment option, ALZET^®^ osmotic pumps were utilized to deliver Api137 in mice in order to achieve constant plasma concentrations. After 3 h of incubation in saline at 37°C (according to the manufacturer’s protocol) and subcutaneous implantation, the pumps were designed to deliver 8 μL peptide solution per hour over 24 h. Animals that received continuously 9.6 and 12.8 mg/kg/h Api137 were significantly protected with survival rates increasing to 33% and 50%, respectively (Supplementary Figure 7). Surprisingly, the highest dose group of 16 mg/kg/h was not similarly efficacious. The plasma levels determined in uninfected animals after 8 h after implantation were inconsistent and not dose-dependent (Supplementary Figure 8). Thus, the priming procedure was checked by analyzing the delivered solution by RP-HPLC indicating a stable flowrate of 8 μL/h after a longer priming period of 6 h for peptide concentrations of 40 and 80 g/L (Supplementary Figure 9).

**FIGURE 4:**
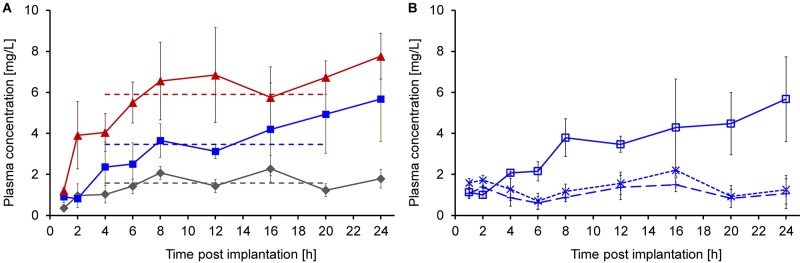
Plasma concentrations of Api137 and its metabolites 1–16 and 1–17 after continuous sc infusion at doses of 6.4 (gray, diamonds), 12.8 (blue, squares), and 19.2 (red, triangles) mg/kg/h in mice (24–32 g, *n* = 9, total = 27) using ALZET^®^ osmotic pumps at a flow rate of 8 μL/h **(A)**. Each data point represents the mean peptide concentration in plasma samples obtained from three individual mice (for sampling scheme see Supplementary Table 1). Plasma levels for all at time points from 1 to 20 h were determined by TNO Triskelion, whereas 24 h samples were measured in house **(A)**. Api137 plasma levels of the medium dose group (12.8 mg/kg/h, blue, open squares) were confirmed and metabolites Api137(1-17) (dotted line, crosses) and Api137(1-16) (dashed line, plusses) were determined in house **(B)**. Pumps were primed using an optimized 6 h priming protocol.

The longer priming period prior to implantation significantly improved the pharmacokinetics of continuously delivered Api137, which appeared in plasma of all animals an hour post implantation (Figure 4A). The plasma levels increased over the next 3 h reaching mean values of 1.6, 3.5, and 5.9 mg/L for doses of 6.4, 12.8, and 19.2 mg/kg/h, respectively (dashed lines). The three dose groups clearly showed dose dependent stable plasma levels between 4 and 20 h, providing evidence of a continuous subcutaneous delivery.

The plasma samples for the medium dose group were reanalyzed in house confirming the previous results. Additional quantification of the two major metabolites Api137(1-17) and (1-16) resulted in stable plasma levels of around 1 mg/L over 24 h (Figure 4B).

### Continuous Subcutaneous Infusion in Infection

**FIGURE 5:**
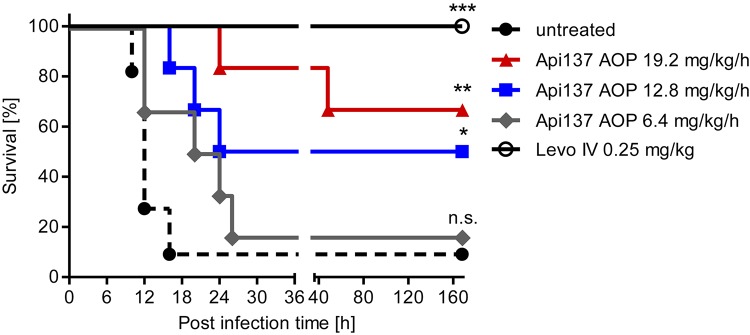
Survival curves obtained for CD-1 mice (20–25 g, total = 36) experimentally infected with *E. coli* ATCC 25922 (6.7 × 10^5^ cfu/mouse) after continuous sc infusion of Api137 using ALZET^®^ osmotic pumps (AOP). Pumps were implanted 1 h post infection to deliver Api137 at doses of 6.4 (gray, diamonds, *n* = 6), 12.8 (blue, squares, *n* = 6), 19.2 mg/kg/h (red, triangles, *n* = 6) with a flow rate of 8 μL/h. Levofloxacin was injected iv at 0.25 mg/kg at 1 h post infection (black, open circles, *n* = 6). Untreated control animals were not subjected to surgery (dotted line, full circles, *n* = 12). Pumps were primed using the optimized 6 h priming protocol. The log-rank test was used for comparison of the survival curves of treated groups to the untreated control. ^∗∗∗^, ^∗∗^, and ^∗^ indicate *p*-values of ≤0.001, ≤0.01, and ≤0.05, respectively; n.s. denotes not significant.

The pumps were utilized again in the infection model to treat animals for 48 h by replacing the first pump after 24 h. The constant delivery of 12.8 and 19.2 mg/kg/h over 48 h improved the survival rates to 50% (3 of 6 mice) and 67% (4 of 6 mice), respectively (Figure 5). The lowest dose of 6.4 mg/kg/h did not provide a significant protection. During the first 40 h, a dose-dependent protection of the animals is evident, although one mouse of the highest dose group showed serious signs of infection and had to be euthanized at this time point. It is possible that the second pump implanted after 24 h did not work properly and triggered a relapse of the infection. Still, the effective dose for 50% survival (ED50) of Api137 in this infection model was calculated as 13.4 mg/kg/h utilizing continuous subcutaneous infusion (Supplementary Figure 10).

## Discussion

Severe multi-drug resistant infections are treated under medical supervision in the hospital using intravenous injection or infusion as preferred administration route for rapid and controlled achievement of efficacious drug plasma levels. Accordingly, the pharmacokinetics of Api137 was evaluated in CD-1 mice and iv and sc administrations yielded maximal plasma levels of 23 and 2.9 mg/L, respectively, correlating well to studies comparing both routes for other peptides including the comparable elimination phases (Kjellev et al., 2007). Unexpectedly, the maximal plasma levels of 6.8 mg/L obtained after iv injection of Api137 at a dose of 5 mg/kg were around 17-fold higher than in a recent study using a similar approach (0.4 mg/L) (Schmidt et al., 2017). Api137 is very rapidly eliminated from the blood stream, i.e., the reported plasma concentrations 5, 20, and 40 min post injection were 0.4, 0.04, and 0.09 mg/L. Here the concentrations were 6.8 mg/L after 5 min and 0.09 mg/L after 30 min. Thus, even slightly different time intervals to collect blood, to prepare plasma, and to freeze the samples might have strong effects on the initial time points. In this respect, the initial plasma concentration obtained 5 min after iv administration should be considered with precaution but is important to judge if the efficacy of Api137 is driven by c_max_ or time over MIC. In comparison, the maximal plasma levels after iv injection of 20 mg/kg here were 6-fold higher than after intraperitoneal administration (3.6 mg/L) in the previous study, whereas, subcutaneous injection of 20 mg/kg yielded almost equal plasma levels. Interestingly, the plasma levels after 30 min dropped to ∼0.5 mg/L independent of the injection route. However, the first *in vivo* study with Api137 relied on an intraperitoneal infection model with the treatment starting 1 h afterwards at the infection site, i.e., i.p., (Schmidt et al., 2017) whereas this study intended to use distinct administration sites iv and sc after the infection was systemically established. Accordingly, four iv doses of 10 and 20 mg/kg increased the survival time of the animals by a few hours but even 20-fold higher doses (iv), compared to the ED_50_ for ip treatment of NMRI mice, did not demonstrate a strong protective effect (Schmidt et al., 2017).

In order to achieve constant Api137 plasma concentrations above MIC for extended time periods we utilized ALZET^®^ mini-osmotic pumps for continuous sc infusion. Priming the filled pumps in saline for a relatively short incubation time of 3 h lead to inconsistent results in pharmacokinetics and the infection model. After this time the osmotic pressure on the reservoir of the pumps was most likely not high enough to achieve constant delivery at 8 μL/h and partially clotting or other effects prevent proper function. However, after priming of the pumps for 6 h the pharmacokinetic analysis showed dose-dependent stable levels after 4 h in the range of ∼6 mg/L for the highest dose, which was slightly above the MIC of Api137 against *E. coli* ATCC 25922 *in vitro* (4 mg/L) (Schmidt et al., 2017). It should be noted that the target concentration level of Api137 is reached only 3 h in total after infection and thus 2 h later compared to single sc injection schemes due to the delayed pump implantation surgery, i.e., only 9 h before the first untreated mice had to be euthanized. Still, the highest dose lead to 67% survival confirming a successful treatment of the intraperitoneal infection using a distinct administration site. Specifically, Api137 was delivered sc by pumps implanted on the back, diffused through the interstitial fluid into the blood before it passed the peritoneum into the intraperitoneal space. Significantly, the therapy did not only control the infection during treatment but allowed the animals to survive the following observation period of 3 days without further antibiotic treatment. Having demonstrated a clear therapeutic effect, it was not attended to optimize the therapy providing 100% survival rates through means such as higher pump capacity or earlier pump implantation. Additionally, it is an improvement to achieve stable plasma levels of ∼6 mg/L by continuous infusion of ∼20 mg/kg per hour whereas a single sc injection with the same dose shows a peak concentration of 2.9 mg/L that has a fast decay to 0.5 mg/L over the next 30 min.

**FIGURE 6:**
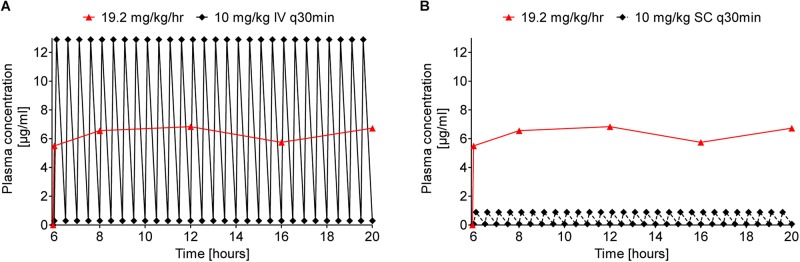
Comparison of predicted Api137 plasma concentration curves for multiple iv (**A**, full line and diamonds) and sc (**B**, dashed line and diamonds) against measured plasma concentration after continuous sc infusion (red line and triangles). The prediction assumes multiple injections every 30 minutes corresponding to a total hourly dose of 20 mg/kg comparable to 19.2 mg/kg delivered hourly by the pump. For calculation of the area under the curve (AUC) with GraphPad Prism 5.02, data points within the period of constant plasma levels (6–20 h post implantation) were included for continuous sc infusion (AUC = 89 μg × min/mL). The AUC for 10 mg/kg q30 min iv and sc were predicted for the same period with 92 and 7 μg × min/mL, respectively.

As an interesting aspect, the area under the curve (AUC) for continuous sc infusion (19.2 mg/kg/h) was calculated for the time of stable plasma levels with 89 μg × min/mL. For comparison, a multiple dosing scheme of single injections every 30 min was predicted leading to a comparable hourly dose of 20 mg/kg, i.e., 10 mg/kg iv and sc, respectively (Figure 6). Taken the plasma levels at 5 min and 30 min post injection into consideration, the AUC was estimated with 92 and 7 μg × min/mL. The good correlation between the AUC of continuous sc infusion and predicted multiple iv injections could indicate that the cleared peptide from the blood stream is continuously replaced by the pump without changing the rate of clearance. The tenfold lower AUC for multiple sc injection could be explained by the delay in the translocation of the peptide from the subcutaneous “depot” via interstitial fluid to the blood stream preventing higher maximum plasma levels after injection.

In the present study, levofloxacin as control was more efficacious, which might be related to (i) starting the treatment one instead of 3 h post infection and (ii) the PK of levofloxacin is more favorable with respect to clearance rates and as a small molecule to enter the peritoneal space, whereas Api137 appears to be partially degraded intraperitoneally (Schmidt et al., 2017). The dose for 100% protection by Api137 (0.6 mg/kg) administered ip 1 h after ip infection was surprisingly low and related to both its local effect at the infection site and a systemic effect before renal clearance. The partial degradation of Api137 after translocation in the intraperitoneal space could also explain the marginal effect of therapies using iv and sc injections and that the maximal plasma concentrations most likely do not translate into high ip concentrations. Therefore, the therapeutic effect of Api137 administered by iv or sc injection might be mostly restricted to pathogens in blood and organs allowing bacteria to constantly spread from the original intraperitoneal infection site into the body when the plasma level of Api137 drops after injection. Continuous sc infusion of Api137 overcomes this limitation as the peptide is constantly delivered to the blood and further to the peritoneum replacing the degraded portion of the compound. Thus, administration routes depend on the infection site and the *in vivo* efficacy of Api137 has to be further investigated by detailed PK-PD studies. Among apidaecin derivatives, Api137 showed the best *in vitro* stability in serum and plasma compared to other PrAMPs in a recent study showing different stability of PrAMPs in fresh blood, serum and plasma (Berthold et al., 2013; Böttger et al., 2017). In light of these recent studies, other proline-rich antimicrobial peptides with higher stability in the intraperitoneal space might be more promising to treat such intraperitoneal infections.

## Data Availability Statement

All datasets generated for this study are included in the manuscript/Supplementary Files.

## Ethics Statement

All animal studies were performed at ViviSource Laboratories, Inc. (Waltham, MA, United States) in compliance with the Animal Welfare Act, the Guide for the Care and Use of Laboratory Animals, and the Office of Laboratory Animal Welfare (approved Institutional Animal Care and Use Committee, IACUC protocols No. VVS12-026 and VVS12-030).

## Author Contributions

DK, KA, and RH contributed to the study design, data evaluation, and the preparation of the manuscript. RS contributed to the in house quantification of Api137 and its metabolites.

## Conflict of Interest

RH and KA are co-founders and shareholders of AMP-Therapeutics GmbH (Leipzig, Germany) and members of its scientific advisory board. DK was a part-time co-worker of AMPT. The remaining author declares that the research was conducted in the absence of any commercial or financial relationships that could be construed as a potential conflict of interest.
