# Supplementary material for: Continuous Subcutaneous Delivery of Proline-Rich Antimicrobial Peptide Api137 Provides Superior Efficacy to Intravenous Administration in a Mouse Infection Model
Source: Front Microbiol. 2019 Oct 2;10:2283. doi: 10.3389/fmicb.2019.02283 (PMC6783563; doi:10.3389/fmicb.2019.02283)
Supplement: Supplementary file 1 [file Data_Sheet_1.PDF]

Supplementary Material

Continuous subcutaneous delivery of proline-rich antimicrobial peptide Api137 provides superior efficacy to intravenous administration in a mouse infection model.

Daniel Knappe, Rico Schmidt, Knut Adermann, and Ralf Hoffmann

Table of contents

1. Quantitative analysis of Api137 in plasma using a LC-MS/ MS method S2

1.1. Sample preparation S2

1.2. RPC-ESI-MS/MS equipment and conditions S3

1.3. UPLC-Gradient S3

1.4. Conditions for multiple-reaction monitoring S3

1.5. Data evaluation S3

Figure S1. Dosing scheme iv PK mice S4

Figure S2. Dosing scheme iv PK rats S4

Figure S3. PK Api137 in rats after iv and sc administration S5

Figure S4. PK Api137 in rats after multiple doses iv administration S5

Figure S5. Ip infection of CD-1 mice with *E. coli* ATCC 25922, two treatments S6

Figure S6. Ip infection of CD-1 mice with *E. coli* ATCC 25922, sc treatment S6

Figure S7. PK Api137 using continuous sc delivery with ALZET® osmotic pumps S7

Figure S8. Ip infection of CD-1 mice with *E. coli* ATCC 25922, ALZET® pump S8

Figure S9. In vitro flow rate of ALZET® osmotic pump S8

Figure S10. Dose-response curve for experiment in Figure 5 S9

Table S1. Blood collection scheme after ALZET® pump implantation S10

Table S2. Survival of CD-1 mice in ip infection with *E. coli* and iv or sc treatment S11

1. Quantitative analysis of Api137 in plasma using a LC-MS/ MS method

Chemicals were obtained from the following manufacturers. Biosolve B.V. (Valkenswaard, The Netherlands): acetonitrile, formic acid, methanol (all ULC/MS grade); Sigma-Aldrich (St. Louis, MO, USA): phosphoric acid (Fluka, 85%), trifluoroacetic acid (Reagent Plus, 99%); Merck KGaA (Darmstadt, Germany): ammonia (25%); water (Milli-Q® Advantage A10 Water purification system)

1.1.Sample preparation

Internal standard solution (10 µL, Api134, 5 µg/mL) was added to a plasma sample (50 µL or 25 µL) and mixed with aqueous phosphoric acid (50 µL or 75 µL; 4%, v/v). Solid phase extraction (SPE) was performed on a WCX micro-elution plate (2 mg, Waters):

SPE step	Solvent	Volume [µL]
Conditioning I	Methanol	200
Conditioning II	Purified water	200
Sample application	Diluted plasma sample	110
Wash step I	Aqueous ammonia (5%, v/v)	200
Wash step II	Aqueous acetonitrile (20%, v/v)	200
Elution I	Aqueous acetonitrile (75%, v/v) including trifluoroacetic acid (1%, v/v)	50
Elution II	Aqueous acetonitrile (75%, v/v) including trifluoroacetic acid (1%, v/v)	50

Water (100 µL) was added to the eluate and the plate was covered to avoid avaporation. The plate was shaken (10 min, plate shaker) and the extract was analysed using RPC-MS/MS.

1.2. RPC-ESI-MS/MS equipment and conditions

Equipment/Parameter	Value
UPLC system	Acquity (Micromass Waters)
UPLC column temperature	50 °C
Autosampler temperature	10 °C
HPLC column	Acquity C18 BEH, 100 x 2.1 mm, 1.7 µm (Waters)
Flow rate	400 µL/min
Mobile phase A	1% formic acid in water
Mobile phase B	1% formic acid in acetonitrile
Strong Wash [Volume]	Methanol/Water/Formic acid (80/20/0.1) [200 µL]
Weak wash [Volume]	Acetonitrile/Water/Trifluoroacetic acid (40/60/0.25) [600 µL]
Injection volume	5 µL
Mass spectrometer	XEVO TQS (Micromass Waters)
Ionization mode	Electrospray ionization (positive mode)
Capillary	0.7 kV
Source temperature	150 °C
Desolvation temperature	600 °C
Cone gas flow	150 L/h
Desolvation gas flow	1000 L/h

1.3. UPLC Gradient

Time [min]	Mobile phase B [%]
2	2
8	25
8.1	80
9.0	80
9.1	2
10.0	2

1.4. Conditions for multiple reaction monitoring

Peptide	Precursor ion (m/z)	Product ion (m/z)	Cone [V]	Collision energy [eV]
Api137	459.3	450.2	40	14
Api134	450.7	530.5	40	12

1.5. Data evaluation

The peak area of the peptides was obtained using MassLynx 4.1 software (Micromass Waters). The peak area ratio (PAR) of peptide Api137 and the internal standard Api134 (Y-axis) was plotted versus the actual peptide concentration (x-axis) to construct a calibration curve.

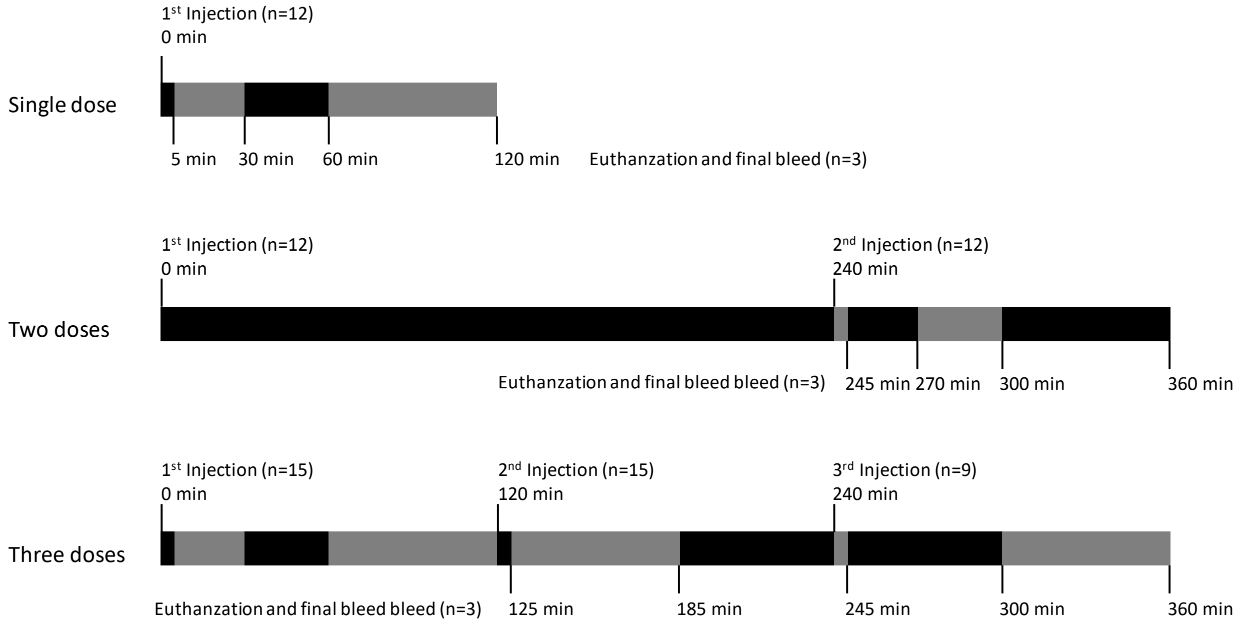

Supplementary Figure 1: Dosing schemes and time-points of blood collection for pharmacokinetic analysis of three mice per dose group and time-point. Three groups of CD-1 mice receiving one dose, two doses, or three doses Api137 were included into the study. For each time-point three animals per group were euthanized and blood was collected via cardiac puncture.

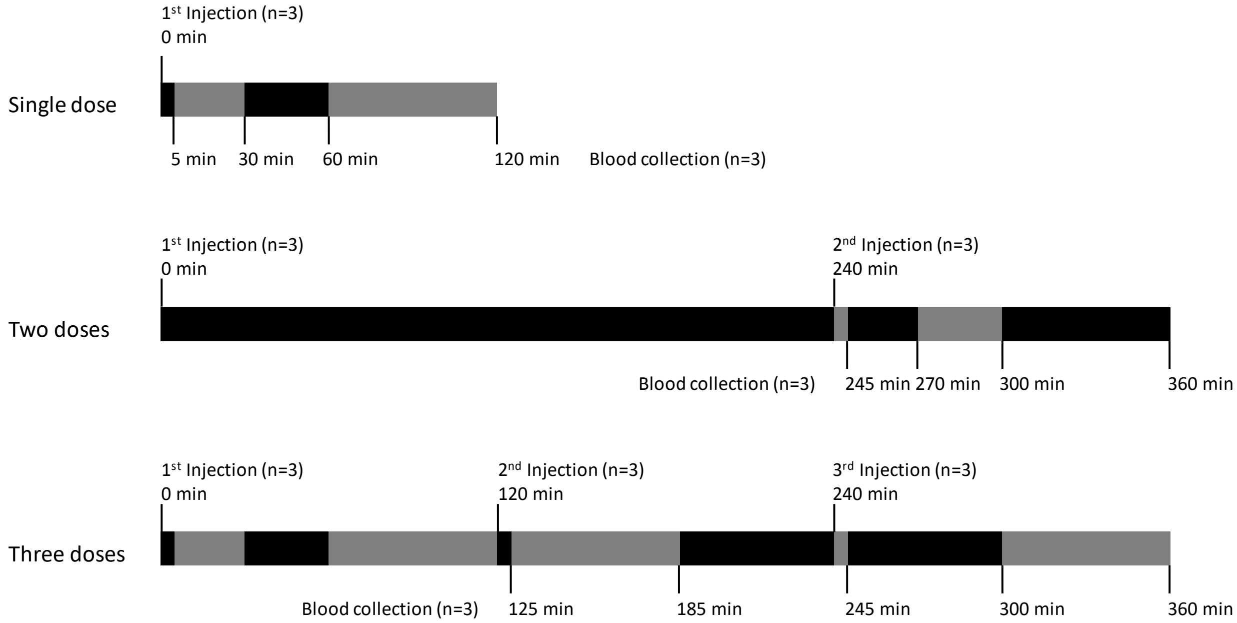

Supplementary Figure 2: Dosing schemes and time-points of blood collection for pharmacokinetic analysis of three rats per dose group. Three groups of SD rats receiving one dose, two doses, or three doses Api137 were included into the study. Blood of three rats were analyzed at each time point.

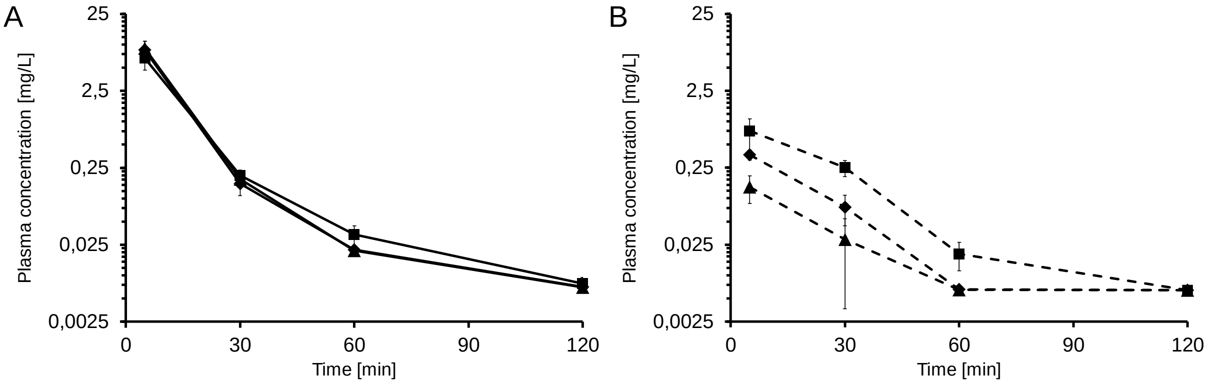

Supplementary Figure 3. Pharmacokinetics of Api137 in rats after single dose iv (A; full lines) and sc (B; dashed lines) injection of 5 (▲), 10 (♦), and 20 mg/kg BW (■), respectively. Each data point represents the mean of peptide concentration in the plasma of three individual animals.

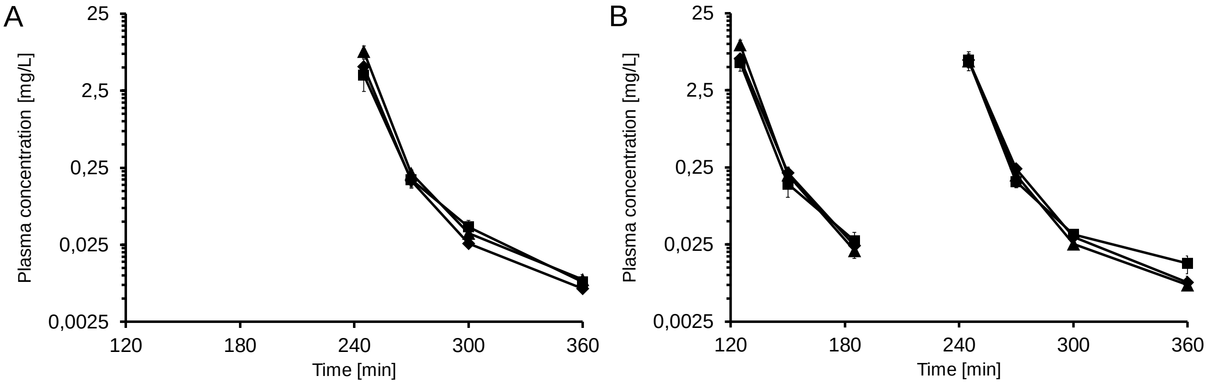

Supplementary Figure 4. Pharmacokinetics of Api137 in rats after two (A; 0 and 240 min) and three (B; 0, 120, 240 min) iv injections at doses of 5 (▲), 10 (♦), and 20 mg/kg BW (■), respectively. Each data point represents the mean of peptide concentration in the plasma of three individual animals.

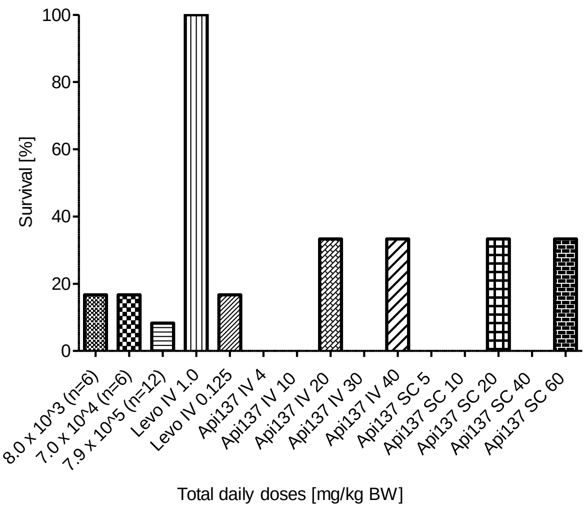

Supplementary Figure 5. Female CD-1 mice (18-20 g) were infected ip with *E. coli* ATCC 25922 (7.9 x 10^5 cfu/mouse). Api137 was administered iv or sc at 1 and 5 hours post infection (n=6). Levofloxacin was administered iv at 1 hour post infection. Survival was determined after 48 hours. The three bars on the left side show the untreated controls infected with three different cfu numbers.

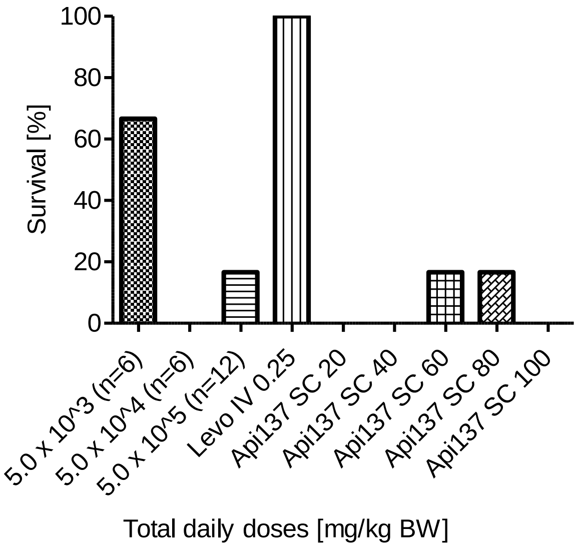

Supplementary Figure 6. Female CD-1 mice (18-20 g) were infected ip with *E. coli* ATCC 25922 (5.0 x 10^5 cfu). Api137 was administered sc at 30, 60, 90, 120 min post infection (n=6). Levofloxacin was administered iv at 30 min post infection. Survival was calculated after 48 hours. The three bars on the left side show the untreated controls infected with three different cfu numbers.

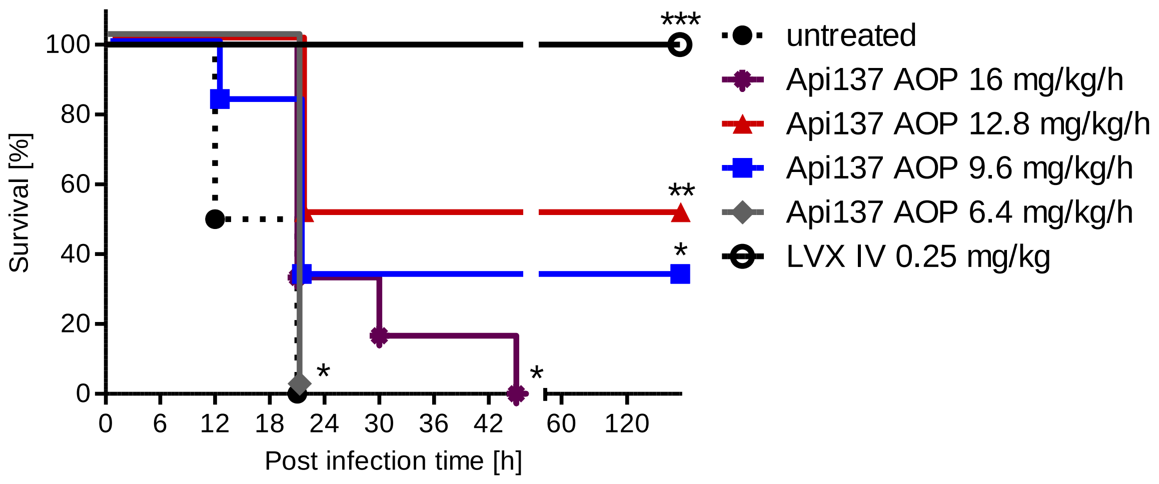

Supplementary Figure 7: Survival curves obtained from experimental infection of CD-1 mice (n=6, 26-32 g) with *E. coli* ATCC 25922 (4.8 x 10^5 cfu/mouse) after continuous sc infusion of Api137 using ALZET® osmotic pumps (AOP). Pumps were implanted 1 h post infection and deliver Api137 at doses of of 6.4 (grey, diamonds), 9.6 (blue, squares), 12.8 (red, triangles), and 16 mg/kg/h (purple, asterisks) with a flow rate of 8 µL/h. Levofloxacin (LVX) was injected iv at 0.25 mg/kg at 1 h post infection (black, open circles). Untreated controls were not subjected to surgery (dotted line, circles). The log-rank test was used for comparison of the survival curves of treated groups to the untreated control. ***, **, and * indicate p-values of $\leq$0.001, $\leq$ 0.01, and $\leq$ 0.05, respectively.

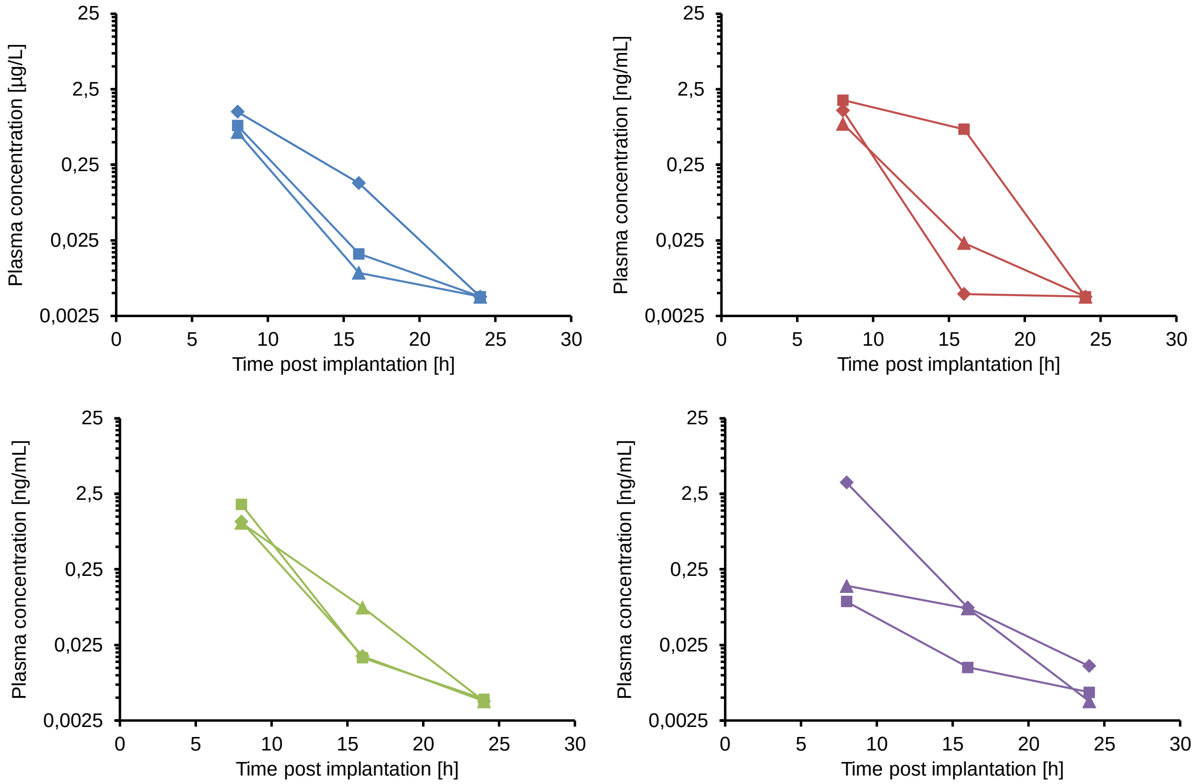

Supplementary Figure 8. Pharmacokinetics of ATA26 after continuous sc infusion of 6.4 (blue), 9.6 (red), 12.8 (green), and 16 (purple) mg/kg/h in mice (n=3, 25-28 g) using Alzet® osmotic pumps. Preliminary experiment with pump priming according to the manufacturers protocol. Pumps were primed for 3 hours. Each curve represents the peptide concentration in the plasma of one animal at 8, 16, and 20 h post implantation.

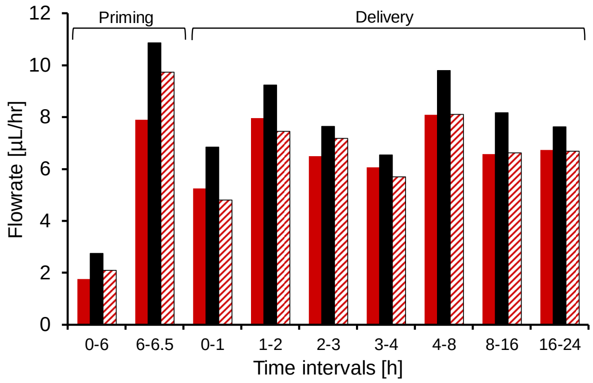

Supplementary Figure 9. Flow rate of ALZET® osmotic pumps containing Api137 in saline (40 and 80 mg/mL, red and black bars, respectively) and Api137 in 0.1% bovine serum albumine in saline (40 mg/mL, striped red bars) after priming (6.5 h). Flowrate was determined after incubation of the filled pumps in saline at 37 °C using reversed phase chromatography.

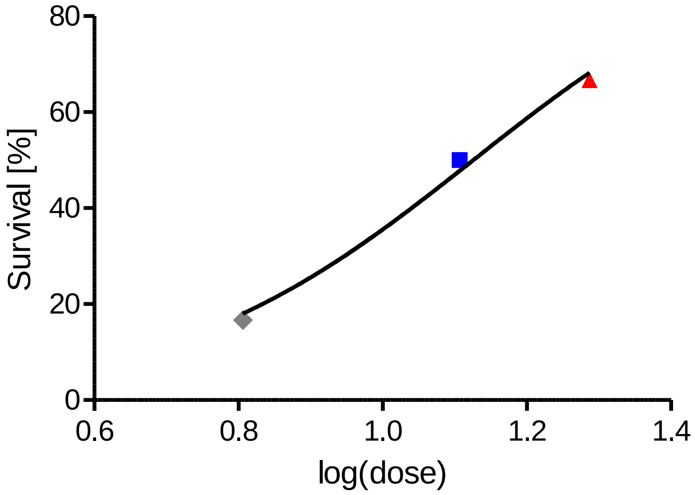

Supplementary Figure 10. Dose-response curve for optimized infection experiment using ALZET® osmotic pumps (Figure 5). The log of doses 6.4 (grey diamond), 12.8 (blue square), and 19.2 mg/kg/h (red triangle) is plotted versus survival. Data is fitted using "dose-response log(inhibitor) vs. normalized response – variable slope" function with "least squares fit" using GraphPad Prism® 5.02. The ED_{50} was calculated with 13.4 mg/kg/h.

Supplementary Table 1: Blood collection scheme after implantation of ALZET® osmotic pumps. The corresponding pharmacokinetics are shown in Figure 4.

Dose group [mg/kg/hr]	Animal No.	Blood Collection (hours post implantation)	Total number of animals
6.4	1, 2, 3	1, 4, 8*	9
	4, 5, 6	2, 12, 16*	
	7, 8, 9	6, 20, 24*	
12.8	11, 12, 13	1, 4, 8*	9
	14, 15, 16	2, 12, 16*	
	17, 18, 19	6, 20, 24*	
19.2	20, 21, 22	1, 4, 8*	9
	23, 24, 25	2, 12, 16*	
	26, 27, 28	6, 20, 24*	

* After 8, 16, and 24 hours, respectively, mice of each group were euthanized by carbon dioxide inhalation and blood was collected via cardiac puncture.

Supplementary Table 2: Survival times and mean survival times obtained from experimental ip infection of CD-1 mice with *E. coli* ATCC 25922 (2.0 x 10^6 cfu/mouse) after treatment with Api137 administered iv or sc.

Animal No.	Untreated	60 mg/kg sc	100 mg/kg sc	10 mg/kg iv	20 mg/kg iv
1	12	12	16	16	16
2	16	16	16	16	16
3	16	16	18	16	16
4	16	22	18	18	20
5	16	22	18	18	26
6	>120*	22	24	24	>120*
mean survival time	15.2	18.3	18.3	18.0	18.8
standard deviation	1.6	3.9	2.7	2.8	3.9

* Animals surviving longer than 120 h are not included in the calculation of the mean survival time.
